# A single migrant enhances the genetic diversity of an inbred puma population

**DOI:** 10.1098/rsos.170115

**Published:** 2017-05-24

**Authors:** Kyle D. Gustafson, T. Winston Vickers, Walter M. Boyce, Holly B. Ernest

**Affiliations:** 1 Wildlife Genomics and Disease Ecology Laboratory, Department of Veterinary Sciences, University of Wyoming, Laramie, WY 82070, USA; 2 Karen C. Drayer Wildlife Health Center, School of Veterinary Medicine, University of California, Davis, CA 95616, USA

**Keywords:** *Puma concolor*, gene flow, genetic rescue, road ecology, conservation genetics, landscape genetics

## Abstract

Migration is essential for maintaining genetic diversity among populations, and pumas (*Puma concolor*) provide an excellent model for studying the genetic impacts of migrants on populations isolated by increasing human development. In densely populated southern California, USA, puma populations on the east and west side of interstate highway 15 (I-15) have become fragmented into a small inbred population on the west side (Santa Ana Mountains) and a relatively larger, more diverse population on the east side (Eastern Peninsular Range). From 146 sampled pumas, genetic analyses indicate seven pumas crossed I-15 over the last 15 years, including four males from west to east, and three males from east to west. However, only a single migrant (named M86) was detected to have produced offspring and contribute to gene flow across the I-15 barrier*.* Prior to the M86 migration, the Santa Ana population exhibited inbreeding and had significantly lower genetic diversity than the Eastern Peninsular Range population. After M86 emigrated, he sired 11 offspring with Santa Ana females, decreasing inbreeding measures and raising heterozygosity to levels similar to pumas in the Eastern Peninsular Range. The emigration of M86 also introduced new alleles into the Santa Ana population, although allelic richness still remained significantly lower than the Eastern Peninsular population. Our results clearly show the benefit of a single migrant to the genetics of a small, isolated population. However, ongoing development and habitat loss on both sides of I-15 will increasingly strengthen the barrier to successful migration. Further monitoring, and potential human intervention, including minimizing development effects on connectivity, adding or improving freeway crossing structures, or animal translocation, may be needed to ensure adequate gene flow and long-term persistence of the Santa Ana puma population.

## 1. Introduction

Without the benefits of immigration, genetic drift and breeding among closely related individuals can lead to an accumulation of deleterious alleles and inbreeding depression, reducing population fitness and increasing extinction risk [1–3]. Immigration benefits populations primarily by increasing heterozygosity and allelic richness, both of which are critical for population persistence [4,5]. Heterozygosity is of immediate importance to individual and population fitness [6,7], whereas allelic richness is directly related to the adaptive potential and long-term viability of populations [8,9]. In small populations, heterozygosity is lost at a slower rate and regained more quickly than allelic richness [2,10,11]. Although a single migrant per generation may be sufficient for maintaining genetic diversity among populations [12–14], populations are becoming increasingly fragmented by human development [15,16], and the desirable one-migrant-per-generation minimum is not always met [12,17–19].

In the United States, fragmented puma (*Puma concolor*) populations are becoming models for the study of genetics in small, isolated populations [20–24]. Complete geographical isolation of the Florida panther (*P. concolor coryi*) resulting in severe inbreeding is the extreme example [25]. Translocations of pumas from Texas to Florida have successfully alleviated inbreeding depression but, without additional immigration (natural or artificial), long-term population viability is uncertain [22,26]. Puma populations along the highly urbanized west coast of the United States are also becoming isolated, primarily by expanding human development [27–31]. Demographic and genetic concerns, along with threat of disease, have brought the long-term viability of these urban puma populations into question, and genetic restoration may be required [21,23,24,32–34].

Within southern California, our research team has been monitoring puma populations in the Santa Ana Mountains and Eastern Peninsular Range since the early 2000s [34]. Pumas in the Santa Ana Mountains are exhibiting signs of inbreeding, and are isolated from the more genetically diverse Eastern Peninsular Range population by an 8–10 lane freeway (Interstate 15 [21,34]), which is one of two major freeways that run north–south between two of the most urban regions in the United States (the greater Los Angeles area: approx. 18.7 million; San Diego County: approx. 3.3 million [35]). In *P. concolor*, young males (approx. 18 months of age) are the primary dispersers [28,36] and are responsible for significant gene flow among populations [37]. Our team previously documented that a single male crossed I-15 from the Eastern Peninsular Range and successfully produced offspring in the Santa Ana Mountains [21,34]. However, the impact of that individual, and potentially others, on the genetic diversity of the inbred Santa Ana population has not been studied.

To assess the effects that migration can have on small, isolated populations, we studied the impact of inter-population movements on inbreeding, heterozygosity and allelic richness between the Santa Ana and Eastern Peninsular Range puma populations [21,34]. We used morphological data from field captures to estimate ages of individual pumas. Assignment and pedigree models were used to determine population and familial structure, and the identification of family units allowed us to more accurately identify migrants and whether they reproduced. We then estimated inbreeding, heterozygosity and allelic richness in the two populations before and after migration events. Our results demonstrate the extent to which a single migrant can benefit the genetics of a small, isolated population.

## 2. Material and methods

### 2.1. Capture methods and age determination

We captured, marked and monitored radio-collared pumas from 2001 to 2016 in the Santa Ana Mountains and Eastern Peninsular Range. Capture methods are detailed in Vickers *et al.* [34]. We examined movements of radio-collared pumas from 2001 to 2016 to help identify potential migrants among ranges. Permission to carry out fieldwork and necessary permits were obtained from the California Department of Fish and Wildlife (CDFW), California Department of Parks and Recreation, The Nature Conservancy, United States (US) Fish and Wildlife Service, US Forest Service, US Bureau of Land Management, US Navy/Marine Corps, Orange County Parks Department, San Diego County Parks Department, Riverside County Parks Department, San Diego State University, University of California–Riverside, Audubon Starr Ranch, Vista Irrigation District, Rancho Mission Viejo/San Juan Company, Sweetwater Authority, California Department of Transportation, the City of San Diego Water Department and Parks Department, and the Irvine Ranch Conservancy as described in Vickers *et al.* [34].

### 2.2. Genetic sampling and microsatellite DNA data collection

We analysed genetic samples from 146 pumas among the Santa Ana Mountains, San Gabriel Mountains, San Bernardino Mountains and the Eastern Peninsular Range. Genetic sampling is detailed in Ernest *et al.* [21]. Briefly, we obtained blood or tissue samples for analysis of nuclear DNA from pumas captured for telemetry studies, and from those found dead or killed by state authorities for livestock depredation or public safety, some preceding year 2001. Forty-four microsatellites (electronic supplementary material, table S1), which met the assumptions of Hardy–Weinberg proportions and linkage equilibria were used for genotyping individuals. Each sample was genotyped at least twice and genotypes were determined by two independent observers. Negative and positive controls were included in each PCR. Specific extraction, PCR and sequencing methods are detailed in Ernest *et al.* [21].

### 2.3. Population assignment

We used spatially explicit Bayesian population assignment programs GENELAND v. 4.0 [38] and TESS v. 2.3 [39] and followed all developer recommendations. In GENELAND, the number of populations (K) is a parameter optimized by the model. First, we ran 15 spatial models from 1 to 10 K. We then ran five spatial models holding K at the modal K of the initial runs. We selected the model with the greatest log posterior probability and ran an admixture model. Each run included an uncertainty on coordinate of 0.01 decimal degrees (approx. 11 km), 100 000 iterations, a thinning interval of 100 and a 25% burn-in period.

In TESS, K must be tested over a range of possible values. First, we ran 10 non-admixture models for each K from 2 to 10. For model comparisons, TESS computes a deviance information criterion (DIC). We ran 10 spatially conditional auto-regressive admixture models for each K to the DIC plateau of non-admixture models. All models included pairwise great circle geographical distances, 100 000 iterations, and a 25% burn-in period. We retained 20% of the models which contained the lowest DIC scores and used CLUMPP v. 1.1.2 [40] to perform model-averaging.

### 2.4. Pedigree reconstruction

We used program CERVUS v. 3.0.7 [41] to construct pedigrees. We ran additional sibship analyses with program COLONY v. 2.0.6.2 [42,43]. In both programs, 1% genotyping error was allowed. Parent–offspring relationships were determined based on age estimates from the morphological data taken during captures.

### 2.5. Measures of genetic diversity and population divergence

To assess inbreeding, we calculated internal relatedness using package Rhh 1.0.2 in program R v. 3.3.0 [44,45]. Internal relatedness measures a relative outbred–inbred continuum, where negative values are suggestive of outbred individuals and positive scores are suggestive of inbreeding [45]. We calculated unbiased expected heterozygosity (*Ĥ*_E_) in GenAlEx 6.502 [46,47]. Unlike observed heterozygosity and measures of allelic richness, *Ĥ*_E_ is a robust genetic diversity estimator for small sample sizes [46,48]. To measure the number of alleles, we calculated allelic richness using sample-size correcting rarefaction methods in FSTAT 2.9.3.2 [49,50]. Population divergence (*F*_ST_) was also calculated in GenAlEx; significance testing was based on 999 permutations [47].

Internal relatedness, heterozygosity and allelic richness data met the assumptions of linear models. Population differences in internal relatedness were calculated using analyses of variance (ANOVAs). To assess the validity of internal relatedness as a measure of inbreeding, we used a *T*-test to determine if the internal relatedness of offspring was higher than the average internal relatedness of their consanguineous parents. Temporal and population differences in heterozygosity and allelic richness were tested using a repeated-measures (RM) ANOVA with locus treated as the repeated measure. We used Tukey's honest significant differences *post hoc* tests. Statistics were computed in program R using base software for linear models (ANOVAs, *T*-tests) and packages lme4 1.1–12 [51] and lsmeans 2.23–5 [52] for mixed models (RM-ANOVAs). For temporal analyses, samples collected from 2000 to 2016 were divided into five time periods (2000–2003, 2004–2006, 2007–2009, 2010–2012, 2013–2016). If an individual puma was estimated to have been alive at any point during that time period, it was included in the analysis.

## 3. Results

### 3.1. Population assignments and migrations

**Figure 1.**
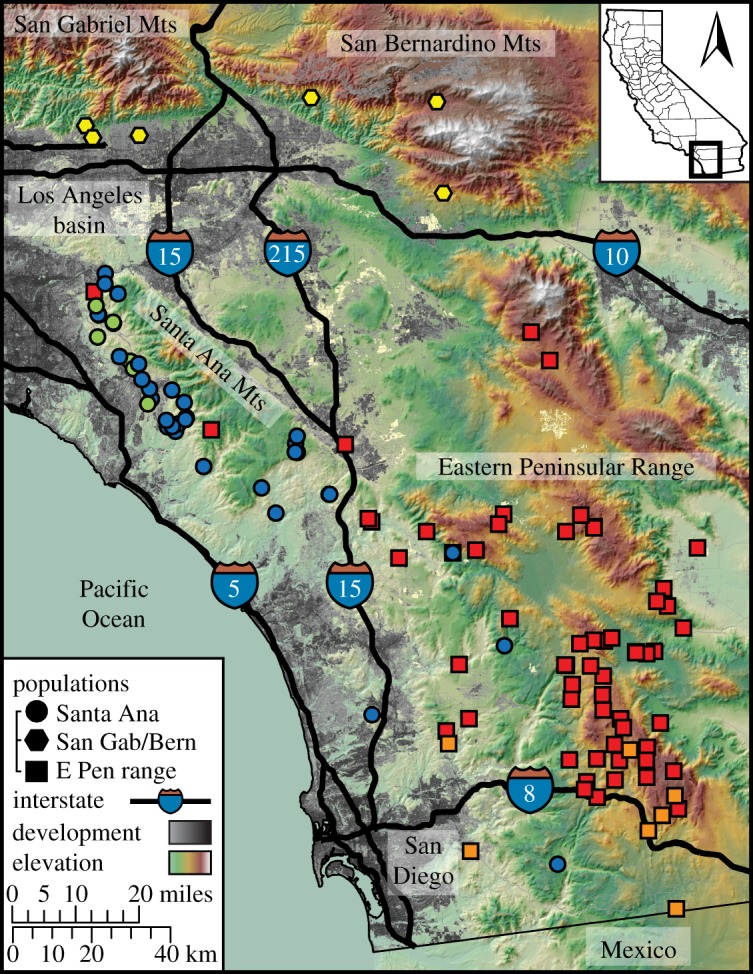
Pumas sampled (*N* = 146) in the Los Angeles–San Diego region of California, USA (inset map) were subdivided into three populations separated by major interstate highways (I-15, I-10). Circles, hexagons and squares indicate pumas, respectively, belonging to the Santa Ana, San Gabriel/Bernardino and Eastern Peninsular Range populations identified in GENELAND (figure 2*a*). Colours correspond to highest subpopulation admixture proportions identified in TESS (figure 2*b*).

**Figure 2.**
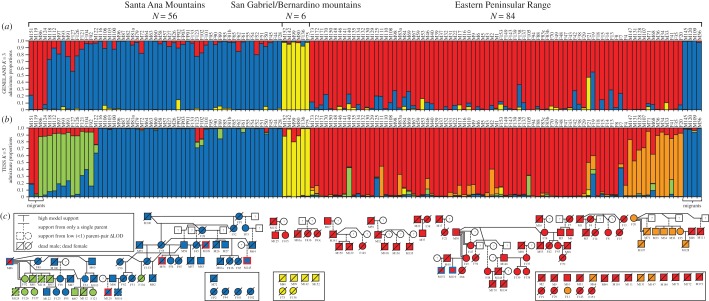
Population assignments (*a*,*b*) and pedigree analyses (*c*) of pumas (*N* = 146) in the Los Angeles–San Diego region of California. Program GENELAND (*a*) identified three populations including the Santa Ana (blue), San Gabriel/Bernardino (yellow) and Eastern Peninsular Range (red) mountains. Program TESS (*b*) identified additional substructure within the Santa Ana population (green) and within the Eastern Peninsular Range (orange). Pumas were numbered in the order they were captured and M or F refers to whether that individual was a male or female, respectively (upper *x*-axis). Migrants from the Eastern Peninsular Range to the Santa Ana Mountains (*N* = 3) are indicated on the left; migrants from the Santa Ana Mountains to the Eastern Peninsular Range (*N* = 4) are indicated on the right. Familial relationships (*c*) were estimated using the pedigree-reconstruction program CERVUS, and confirmed with program COLONY. Within the pedigree, individual squares (males) and circles (females) are filled based on maximum admixture proportion in TESS (*b*). Coloured borders indicate the location where immigrant pumas were sampled. Individuals with unknown lineages (i.e. singletons) are pooled in open boxes. Instances of inbreeding are indicated with double lines.

Based on our Bayesian population assignment analyses, we identified three geographically structured populations (Santa Ana, SA; San Gabriel/Bernardino, SGB; Eastern Peninsular Range, EP) with additional substructure within SA and EP (figures 1 and 2). Given the low sample size of SGB pumas (*N* = 6), temporal measures of internal relatedness, heterozygosity and allelic richness could not be calculated and further analyses were restricted to SA and EP. Pedigree analyses indicated the additional substructure within SA was composed of a male migrant (named M86; M/F for male/female, followed by capture number) and his 11 offspring (figure 2*b*,*c*). Substructure within EP primarily corresponded to a female puma (F20) and her offspring, but several substructure-assigned individuals were not first-order relatives (i.e. parent–offspring, full-sib) of F20 (figure 2*b*,*c*).

With our pedigree analyses, we identified frequent mating within, but not among, populations. There was only one observed instance of mating among populations, which occurred when M86 migrated from EP into the Santa Ana Mountains in 2010 and mated with F61, F89, F133 and mated with his offspring F92 (figure 2). Notably, F92 had the lowest internal relatedness of all pumas sampled in the Santa Ana Mountains (−0.14). Additional detections of inbreeding in EP included M39 mating with his daughter F47 to produce F49, with whom he also mated. M71 also mated with his mother, F20.

**Figure 3.**
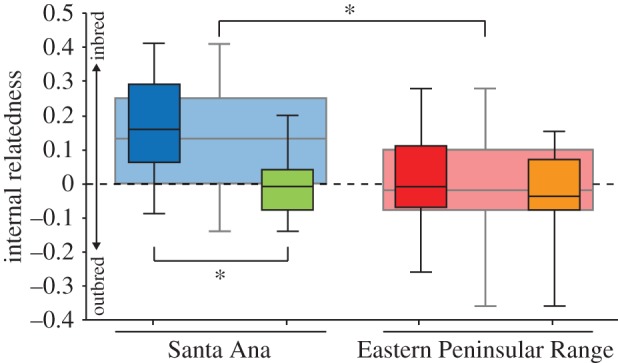
Internal relatedness (i.e. relative outbred–inbred continuum) of Santa Ana and Eastern Peninsular Range pumas. Wide background boxplots represent populations identified in GENELAND (figure 2*a*). Colours of foreground boxes correspond to TESS subpopulations (figure 2*b*). Green corresponds to M86 and his offspring. Orange primarily corresponds to F20 and her offspring. Min, max, inter-quartiles and median are presented.

Internal relatedness was significantly (*T*_137_ = 5.4, *p* < 0.001) higher in SA (mean ± s.e.: 0.13 ± 0.02) than EP (0.00 ± 0.01; figure 3). Inbred offspring had significantly higher internal relatedness relative to the average internal relatedness of their consanguineous parents (+Δ0.18 ± 0.05; *T*_10_ = 3.79, *p* = 0.003).

### 3.2. Migration effects on population genetics

Our genetic analyses detected a total of seven migrants across I-15, all of which were males (figure 2). The three migrants originating from EP had negative internal relatedness (M86: −0.07, M119: −0.07, M151: −0.18), indicative of outbreeding. All but one of four migrants from SA had positive internal relatedness (M56: 0.29, M109: −0.09, M120: 0.38, M145: 0.17). M86 was the only migrant known to reproduce, with a total of 11 offspring identified (figure 2*c*). M86 and his offspring had significantly lower internal relatedness compared with other SA pumas (ANOVA *F*_3,135_ = 16.87, *p* < 0.001; Tukey's HSD *p* = 0.007; figure 3). All other subpopulation pairwise comparisons were not significant (Tukey's HSD *p* > 0.8), indicating M86 decreased internal relatedness of his SA-offspring to comparable levels of EP pumas (figure 3).

**Figure 4.**
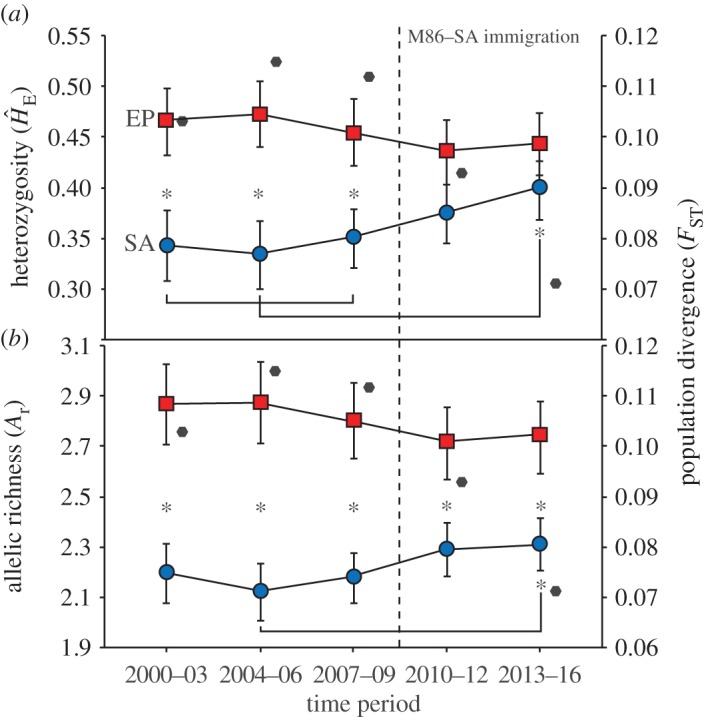
Temporal patterns of (*a*) unbiased expected heterozygosity, (*b*) allelic richness, and population divergence (*F*_ST_: black hexagons; right *y*-axis) for the Eastern Peninsular Range (EP) and Santa Ana (SA) puma populations before (left of vertical dashed line) and after (right of the vertical dashed line) the migration of M86 into SA from EP. Unbiased expected heterozygosity is a robust genetic diversity estimator for small sample sizes; allelic richness was sample-size corrected. All pairwise *F*_ST_ estimates were significantly different from zero. Squares/circles are offset from hexagons for easier visualization. Pumas estimated to be alive at any point within time periods were included in time period estimates. Means and s.e.s are presented. Sample sizes include 27 (number of males & females: 13 & 14, respectively), 25 (11 & 14), 26 (16 & 10), 28 (22 & 6), and 33 (26 & 7) for EP; 10 (1 & 9), 9 (5 & 4), 29 (16 & 13), 38 (21 & 17) and 24 (13 & 11) for SA.

Prior to the M86 migration into the Santa Ana Mountains, SA had significantly lower heterozygosity (*Ĥ*_E_) and allelic richness (*A*_r_) than EP in all time periods (RM-ANOVA: Tukey HSD *p* < 0.0001). After the M86 migration, *Ĥ*_E_ increased in SA and was no longer significantly different from EP (*p* = 0.24, *p* = 0.67; respectively). Although *A*_r_ in SA significantly increased after the M86 immigration, SA had significantly lower *A*_r_ than EP in all time periods (*p* < 0.001). SA–EP population divergence (*F*_ST_) decreased after the M86 immigration; however, populations remained significantly diverged at all time periods (999 permutation tests, *p* < 0.001; figure 4).

## 4. Discussion

### 4.1. Single-migrant genetic restoration

Our results demonstrate that a single migrant can have immediate positive effects on the genetics of a small, isolated and inbred puma population. Within the urbanized Los Angeles–San Diego region of California, the Santa Ana Mountains (SA) population is inbred and genetically depauperate compared to the Eastern Peninsular Range (EP) population [21]. Our analyses show that prior to the successful immigration and reproduction by a single male (M86), the SA population had significantly higher internal relatedness, significantly lower heterozygosity and significantly lower allelic richness than the EP population. After the immigration of M86, we observed significant improvements in all genetic measurements. M86 produced 11 offspring with four SA females. Even though one of M86's matings was with his daughter, he and all his offspring had significantly lower internal relatedness (i.e. they were less inbred) than other SA pumas and they exhibited internal relatedness values comparable with EP pumas. Heterozygosity and allelic richness both significantly increased in the SA population after the M86 immigration. Heterozygosity increased to the point that it was no longer significantly different from the EP population. Allelic richness increased more modestly, remaining significantly lower than the EP population in all time points.

It is possible that the emigration of M86 has resulted in genetic rescue (i.e. alleviation of inbreeding depression). However, genetic rescue is difficult to confirm given the natural variation in individual survival/reproduction rates, population dynamics, and environmental conditions which affect individuals and populations [53]. Pumas in the Santa Ana Mountains have low annual survival rates (mean 0.56; [34]), and five of the seven pumas that have crossed I-15 are known to be deceased. Pumas in the region can legally be shot for depredation events (such as when lions are suspected of killing domestic animals or endangered species), are hit by cars, are killed illegally, die of other causes or have limited food resources [34]. Additionally, young immigrant males have the additional challenge of establishing territories, avoiding fights with conspecifics, finding food and mating in a previously unknown landscape [34]. Thus, the migration of two or more EP population pumas per generation is probably needed for successful reproduction at least once per generation and for maintaining genetic diversity in the SA population.

The significant increase in heterozygosity and allelic richness caused by the emigration of M86 suggests that little or no gene flow occurred between the SA and EP populations in the period immediately prior to this study. Although low sample sizes through 2000–2006 in SA could have biased genetic diversity estimates, the low sample size estimates (i.e. 2000–2003 and 2004–2006) were consistent with each other and with the more-intensively sampled time period prior to M86 (i.e. 2007–2009). Additionally, our estimates of genetic diversity are robust to differences in sample size. Given the high mortality rates in the SA, it is remarkable that M86 produced a large number of offspring (*N* = 11) prior to his death. However, his contribution to gene flow was foreshortened because he was killed by a vehicle strike, and over half of his offspring are either deceased or in captivity. Another migrant from EP to SA (M151) was apparently killed prior to successfully siring offspring in the SA. This puma had the lowest internal relatedness of any migrant pumas we sampled, but he was unable to enhance genetic diversity in the SA because he was legally shot on a depredation permit after repeatedly preying on domestic animals. Further monitoring of the only remaining migrant (M119) in the Santa Ana Mountains, as well as M86's surviving offspring, will be important for tracking population viability, and assessing the long-term impacts of migration.

In a region north of the Santa Ana Mountains and west of Los Angeles, pumas in the Santa Monica Mountains exist in very low numbers (approx. 10 total pumas at any one time) and are also isolated by a major roadway [23,27]. Over 10 years, a single male migrant was detected to have crossed the road into the Santa Monica Mountains and was the only detected breeding male. Although that male enhanced genetic diversity after producing eight detected offspring, the genetic structure of pumas in the area were completely changed because of low census size. By contrast, M86 increased genetic diversity of SA despite the Santa Ana Mountains supporting multiple breeding males and approximately 20–30 adult pumas at any one time [54]. Pumas in the Santa Ana Mountains are thought to represent a genetically distinct population [21,34]. Thus, our observations differ from the Santa Monica Mountains, which may represent family- or group-level dynamics. The only reported puma population with a lower heterozygosity than the SA population was in Florida, where pumas nearly went extinct from inbreeding depression, but were genetically rescued by translocating pumas from the state of Texas [22,26]. Given that the SA population has similar estimates of genetic diversity to just a few established pumas in the Santa Monica Mountains [23], our results indicate genetics may be an issue for SA population viability in the near future [24]. The SA population and pumas in the Santa Monica Mountain region are genetically distinct and exhibit no gene flow [21], further illustrating how urbanization and road fragmentation can completely separate populations that are geographically close (less than 70 km in this case). This may indicate a widespread issue for pumas throughout the rapidly urbanizing state of California.

### 4.2. Importance of genetic diversity in small populations

The loss of rare alleles and accumulation of deleterious alleles can occur in small populations through genetic drift and inbreeding [2,55,56]. We used internal relatedness, an individual index ranging from outbred to inbred, to estimate degree of inbreeding [45]. In other mammalian systems, high internal relatedness (indicative of inbreeding) correlates with high incidence of disease [57], low reproductive success [45,58] and low survival [59]. Inbreeding depression is well documented in the Florida puma population and associated physical abnormalities included kinked tails [22,60]. Anecdotal evidence of two SA pumas with the highest internal relatedness values also exhibited kinked tails [21], suggesting inbreeding depression may also be present in the SA population. In this study, internal relatedness increased in offspring produced from consanguineous (i.e. highly related) mate-pairs, validating its use as a measure of inbreeding [45]. However, the observed decrease in internal relatedness does not confirm that genetic rescue has occurred [61], and further studies are needed to explicitly assess the extent of inbreeding depression in the SA population [62].

Heterozygosity is a classic measure of genetic diversity and has been correlated to disease resistance [63,64], resistance to toxicants [65], increased fecundity [66,67] and puma survival [68,69]. We observed a significant increase in heterozygosity after the immigration of M86, to the point that heterozygosity of SA pumas was no longer significantly different from that of EP pumas. Whereas heterozygosity is thought to be of immediate importance to individual and population fitness [2], allelic richness is thought to be more important for the adaptive potential of populations because alleles are the raw material on which evolution occurs [2,9,70]. We observed a significant increase in allelic richness after the immigration of M86 into the SA population. However, allelic richness remained significantly lower than that of the EP population. Larger populations have a greater capacity for unique alleles simply because they have more individuals to harbour them [2,70]. Thus, we do not expect the SA population, which has a smaller effective population size and less habitat [21,34], to ever exhibit the same allelic richness as the EP population.

### 4.3. Implications and conclusion

We identified three puma populations in this region, including the SA, SGB and EP populations. Although population assignment models GENELAND [38,71] and TESS [39,72] showed similar patterns, TESS identified genealogical substructure not identified by GENELAND. We determined the additional substructure in the SA population originated from an immigrant male (M86) and his 11 offspring. However, the additional substructure in the EP population appears to be a family-unit admixed from a currently unsampled population, perhaps from Mexico or Arizona. The SGB population, and the within-population family groups in SA and EP, were not previously identified [21], probably due to limited sampling. Future researchers should consider the behaviour of certain Bayesian clustering algorithms when analysing population genetic structure of populations containing related individuals [73,74]. Without pedigree or relatedness data, family-unit genetic clusters could mistakenly be classified as populations [75].

We identified a total of seven migrants, all males. Four moved east and three moved west across I-15. With the exception of M56 who was GPS-collared when he crossed I-15 in 2010, it is not known exactly when these migrants crossed the I-15 barrier. If they migrated at their dispersal ages, which is most likely, then based on their estimated ages when sampled their crossings would have all occurred between 2008 and 2014. TESS underestimated the number of migrants by including M86 into a SA subpopulation, whereas GENELAND overestimated the number of migrants by incorrectly assigning an inbred offspring (M124) of M86 to the EP population. The addition of our pedigree analysis allowed us to more accurately identify migrants and family groups, and showed that only one of three migrants into the SA population successfully established a territory and mated. Interstates, including I-15, have been reported to be major barriers to puma movement [21,28,34,76], and we suspect I-10 is also a barrier to puma gene flow. However, additional samples and further monitoring in the San Gabriel and San Bernardino Mountains are needed to assess the impact of I-10 on puma movements and population genetics.

Despite the increases in heterozygosity and allelic richness in the SA population, and despite being only separated by a single interstate highway, the SA and EP populations remain significantly diverged (i.e. significant pairwise *F*_ST_), and SA pumas still face threats from disease, human development, and stochastic demographic, genetic and environmental events [24,33,34,77]. As observed in other systems where a single or a few migrants genetically restored a population [14,23,53,78,79], genetic diversity will decrease and inbreeding will increase, without continuous gene flow [22,26,62,80–82]. Our results clearly show the benefit of a single migrant to the genetics of a small, isolated population. However, if one successfully breeding migrant per generation is required for long-term persistence [12,13], multiple migrants (usually dispersing males) must cross I-15 east to west in each generation to presume one is successful at breeding. Thus, future monitoring, and potential human intervention in the form of improved or new I-15 crossing structures, limitations on new development, or puma translocations, may be needed to ensure adequate gene flow and population viability.

The ability of pumas to cross I-15 is very limited, and will only decrease. Several pumas have been killed on I-15 in the past 30 years, but road mortalities are only one aspect of barrier effects. Other barrier effects include the combination of noise, light, human presence, adjacent development and other anthropogenic factors. It is essential that the ability of pumas to cross over or under I-15 not be reduced further, and mitigation measures to reduce road barrier effects should be pursued. As of this writing, large residential developments are proposed to be constructed within the two primary puma travel corridors between the Eastern Peninsular and Santa Ana Ranges, and immediately adjacent to the existing crossing structures [73–86]. Construction of these developments is likely to further degrade the ability of pumas, especially dispersing males who are essential for gene flow, to move between the Eastern Peninsular and Santa Ana Mountain Ranges. The critical importance of successful migration and reproduction to the long-term persistence of the SA population should be considered in the planning and approval process for any development near these key crossing points. The construction of new I-15 crossing structures between the Eastern Peninsular and Santa Ana Mountain Ranges has been under study and discussion by multiple regional governmental entities and various researchers and stakeholders for more than two decades. However, no engineering studies have been done, costs are expected to be substantial and funding sources have not been identified.

These results, in combination with the challenges of preserving and improving the state of genetic connectivity for pumas in this region, emphasize that despite M86's success in improving some genetic parameters of the SA population, the population remains at risk of further genetic decline. The political and conservation barriers are large, and positive change will depend on a great deal of political-will, and both public and private investments. In the case of the Florida panther and the Santa Monica Mountains pumas, large amounts of funding were mobilized once these subpopulations were threatened with extirpation. The SA population, and possibly other puma populations in California in the future, may also need human intervention to persist.

## Supplementary Material

Table S1

## Data accessibility

Data analysed in this paper are accessible through Dryad: (http://dx.doi.org/10.5061/dryad.1kh2n) [87].
