# Supplementary material for: A single migrant enhances the genetic diversity of an inbred puma population
Source: R Soc Open Sci. 2017 May 24;4(5):170115. doi: 10.1098/rsos.170115 (PMC5451821; doi:10.1098/rsos.170115)
Supplement: Table S1 [file rsos170115supp1.docx]

A single migrant enhances the genetic diversity of an inbred puma population

*Royal Society Open Science*

**Table S1.** Microsatellite locus-specific details.; Na: number of alleles; Ne: effective number of alleles; I: Shannon index; H_O_: observed heterozygosity; uH_E_: unbiased expected heterozygosity; F: fixation index.

| Locus | Citation | Na | Ne | I | H_O_ | uH_E_ | F |
| --- | --- | --- | --- | --- | --- | --- | --- |
| A106 | [1] | 2 | 1.382 | 0.449 | 0.317 | 0.277 | -0.146 |
| A208 | [1] | 3 | 1.797 | 0.693 | 0.482 | 0.445 | -0.087 |
| A216 | [1] | 3 | 1.515 | 0.631 | 0.309 | 0.341 | 0.090 |
| A339 | [1] | 4 | 2.031 | 0.899 | 0.424 | 0.509 | 0.164 |
| B003 | [1] | 3 | 2.125 | 0.892 | 0.597 | 0.531 | -0.128 |
| B010 | [1] | 5 | 3.072 | 1.220 | 0.734 | 0.677 | -0.088 |
| B203 | [1] | 4 | 1.372 | 0.546 | 0.245 | 0.272 | 0.099 |
| B207 | [1] | 8 | 4.913 | 1.751 | 0.715 | 0.799 | 0.102 |
| B210 | [1] | 4 | 2.425 | 1.064 | 0.525 | 0.590 | 0.106 |
| C010 | [1] | 4 | 2.297 | 0.988 | 0.482 | 0.567 | 0.146 |
| C108 | [1] | 2 | 1.967 | 0.685 | 0.381 | 0.493 | 0.224 |
| C112 | [1] | 4 | 1.436 | 0.592 | 0.317 | 0.305 | -0.042 |
| C209 | [1] | 2 | 1.955 | 0.682 | 0.489 | 0.490 | -0.001 |
| C217 | [1] | 2 | 1.437 | 0.482 | 0.259 | 0.305 | 0.148 |
| D012 | [1] | 2 | 1.130 | 0.230 | 0.122 | 0.115 | -0.065 |
| D103 | [1] | 5 | 2.110 | 0.984 | 0.439 | 0.528 | 0.166 |
| D217 | [1] | 3 | 2.700 | 1.046 | 0.612 | 0.632 | 0.029 |
| FCA008 | [2] | 2 | 1.014 | 0.043 | 0.014 | 0.014 | -0.007 |
| FCA023 | [2] | 2 | 1.247 | 0.350 | 0.194 | 0.199 | 0.020 |
| FCA026 | [2] | 3 | 2.281 | 0.952 | 0.612 | 0.564 | -0.089 |
| FCA030 | [2] | 6 | 1.862 | 0.893 | 0.441 | 0.465 | 0.047 |
| FCA035 | [2] | 2 | 1.052 | 0.118 | 0.051 | 0.050 | -0.026 |
| F037 | [2] | 5 | 3.179 | 1.233 | 0.619 | 0.688 | 0.097 |
| FCA043 | [2] | 4 | 2.280 | 0.942 | 0.338 | 0.563 | 0.398 |
| FCA052 | [2] | 3 | 2.267 | 0.922 | 0.540 | 0.561 | 0.035 |
| FCA057 | [2] | 4 | 3.990 | 1.385 | 0.784 | 0.752 | -0.046 |
| FCA077 | [2] | 2 | 1.763 | 0.624 | 0.388 | 0.434 | 0.102 |
| FCA078 | [2] | 2 | 1.825 | 0.645 | 0.518 | 0.454 | -0.146 |
| FCA081 | [2] | 5 | 2.356 | 1.130 | 0.504 | 0.578 | 0.125 |
| FCA082 | [2] | 4 | 3.063 | 1.246 | 0.590 | 0.676 | 0.124 |
| FCA090 | [2] | 3 | 1.892 | 0.784 | 0.475 | 0.473 | -0.007 |
| FCA096 | [2] | 3 | 1.266 | 0.435 | 0.230 | 0.211 | -0.096 |
| FCA126 | [2] | 4 | 2.016 | 0.869 | 0.496 | 0.506 | 0.015 |
| FCA132 | [2] | 5 | 3.608 | 1.420 | 0.669 | 0.725 | 0.074 |
| FCA144 | [2] | 3 | 1.473 | 0.608 | 0.317 | 0.322 | 0.014 |
| F146 | [2] | 2 | 1.927 | 0.674 | 0.403 | 0.483 | 0.163 |
| FCA149 | [2] | 2 | 1.059 | 0.130 | 0.058 | 0.056 | -0.030 |
| FCA176 | [2] | 4 | 1.882 | 0.832 | 0.381 | 0.470 | 0.186 |
| FCA221 | [2] | 5 | 2.767 | 1.249 | 0.655 | 0.641 | -0.025 |
| FCA229 | [2] | 3 | 1.228 | 0.390 | 0.201 | 0.186 | -0.085 |
| FCA254 | [2] | 5 | 1.448 | 0.660 | 0.259 | 0.311 | 0.163 |
| FCA290 | [2] | 5 | 2.953 | 1.226 | 0.647 | 0.664 | 0.021 |
| FCA391 | [2] | 6 | 3.065 | 1.257 | 0.583 | 0.676 | 0.135 |
| FCA424 | [2] | 3 | 2.312 | 0.927 | 0.568 | 0.569 | -0.002 |

[1] Kurushima JD, Collins JA, Well JA, Ernest HB. 2006 Development of 21 microsatellite loci for puma (*Puma concolor*) ecology and forensics. *Mol. Ecol. Notes* **6**, 1260-1262. (doi:10.1111/j.1471-8286.2006.01508.x).

[2] Menotti-Raymond M, David VA, Lyons LA, Schäffer AA, Tomlin JF, Hutton MK, O'Brien SJ. 1999 A genetic linkage map of microsatellites in the domestic cat (*Felis catus*). *Genomics* **57**, 9-23. (doi:10.1006/geno.1999.5743).
